# The lobular neoplasia enigma: management and prognosis in a long follow-up case series

**DOI:** 10.1186/s12957-021-02182-w

**Published:** 2021-03-18

**Authors:** Jasna Metovic, Simona Osella Abate, Fulvio Borella, Elena Vissio, Luca Bertero, Giovanna Mariscotti, Manuela Durando, Rebecca Senetta, Ada Ala, Chiara Benedetto, Anna Sapino, Paola Cassoni, Isabella Castellano

**Affiliations:** 1 Department of Oncology, Pathology Unit, University of Turin, Via Santena 7, 10126 Turin, Italy; 2 Department of Medical Sciences, University of Turin, Via Santena 7, 10126 Turin, Italy; 3 Department of Surgical Sciences, Gynecology and Obstetrics 1, University of Turin, Via Ventimiglia 3, 10126 Turin, Italy; 4 Department of Diagnostic Imaging and Radiotherapy, Radiology Institute, University of Turin, Via Genova 3, 10126 Turin, Italy; 5 Breast Surgery Unit, Department of General and Specialistic Surgery, AOU Città della Salute e della Scienza, Turin, Italy; 6 Pathology Division, Candiolo Cancer Institute, FPO-IRCCS, Str. Prov. 142, 10060 Candiolo, Italy

**Keywords:** Breast, Lobular neoplasia, Follow-up, Upgrade, Treatment

## Abstract

**Background:**

Many oncologists debate if lobular neoplasia (LN) is a risk factor or an obligatory precursor of more aggressive disease. This study has three aims: (i) describe the different treatment options (surgical resection vs observation), (ii) investigate the upgrade rate in surgically treated patients, and (iii) evaluate the long-term occurrences of aggressive disease in both operated and unoperated patients.

**Methods:**

A series of 122 patients with LN bioptic diagnosis and follow-up information were selected. Clinical, radiological, and pathological data were collected from medical charts. At definitive histology, either invasive or ductal carcinoma in situ was considered upgraded lesions.

**Results:**

Atypical lobular hyperplasia (ALH), lobular carcinoma in situ (LCIS), and high-grade LN (HG-LN) were diagnosed in 44, 63, and 15 patients, respectively. The median follow-up was 9.5 years. Ninety-nine patients were surgically treated, while 23 underwent clinical-radiological follow-up. An upgrade was observed in 28/99 (28.3%). Age ≥ 54 years (OR 4.01, CI 1.42–11.29, *p* = 0.009), Breast Imaging-Reporting and Data System (BI-RADS) categories 4–5 (OR 3.76, CI 1.37–10.1, *p* = 0.010), and preoperatory HG-LN diagnosis (OR 8.76, 1.82–42.27, *p* = 0.007) were related to upgraded/aggressive disease. During follow-up, 8 patients developed an ipsilateral malignant lesion, four of whom were not initially operated (4/23, 17%).

**Conclusions:**

BI-RADS categories 4–5, HG-LN diagnosis, and age ≥ 54 years were features associated with an upgrade at definitive surgery. Moreover, 17% of unoperated cases developed an aggressive disease, emphasizing that LN patients need close surveillance due to the long-term risk of breast cancer.

**Supplementary Information:**

The online version contains supplementary material available at 10.1186/s12957-021-02182-w.

## Introduction

The term lobular neoplasia (LN) encompasses a group of atypical epithelial lesions originating from the terminal duct lobular unit (TDLU) of the breast. These lesions are characterized by the proliferation of atypical discohesive small uniform cells that do not infiltrate the mammary gland basement membrane [1]. They are traditionally described depending on the degree of involvement of the TDLU acinar structures, from atypical lobular hyperplasia (ALH) to lobular carcinoma in situ (LCIS). Although infrequent, some aggressive variants of LCIS, such as LCIS with florid appearance (FLCIS) and pleomorphic LCIS (PLCIS), may occur. To emphasize that LN actually represents a risk factor rather than an obligatory precursor of more aggressive diseases, the WHO proposed the term lobular intraepithelial neoplasia (LIN) and a three-tiered grading system, LIN1 (corresponding to ALH), LIN2 (LCIS), and LIN3 (PLCIS or FLCIS) in 2003 [2, 3]. However, both the 2012 and the 2019 WHO Editions [4, 5] abandoned the LIN classification, and the traditional categorization was recommended again in routine practice, generating confusion in clinical management. In addition, the 2006 European Guidelines for breast cancer (BC) screening and diagnosis [6] classified preoperative LN as B3, meaning “lesion with uncertain malignant potential,” and reserved B5a (carcinoma in situ) for PLCIS or FLCIS. This issue was further complicated by the latest American Joint Committee on Cancer (AJCC) [7] that, in opposition to the latest Union for International Cancer Control (UICC) TNM Classification [8], avoids “pTis” staging in definitive surgery cases of LN.

Currently, surgical intervention is generally recommended in cases of radio-histological disagreement or when high-grade LN (HG-LN), such as PLCIS or FLCIS, is diagnosed at biopsy [6]. In all the other cases, observation with interval breast imaging is favored as a reasonable alternative [9]. Nevertheless, Bodian et al. [10] showed that the risk of consecutive intraductal carcinoma in LN remains high for at least 20 years. Similarly, another study found a high 10-year incidence (> 7%) of invasive BC after LCIS diagnosis [11].

In this complex background, we would like to describe our hospital’s experience on the management and outcome of a series of patients with a preoperative diagnosis of LN on core needle biopsy (CNB) or vacuum-assisted breast biopsy (VABB). This study has three aims: (i) describe the different treatments of these lesions (surgical resection vs observation), (ii) investigate the upgrade rate in surgically treated patients, and (iii) evaluate the long-term occurrences of aggressive disease in operated and unoperated patients.

## Materials and methods

### Study population

**Fig. 1:**
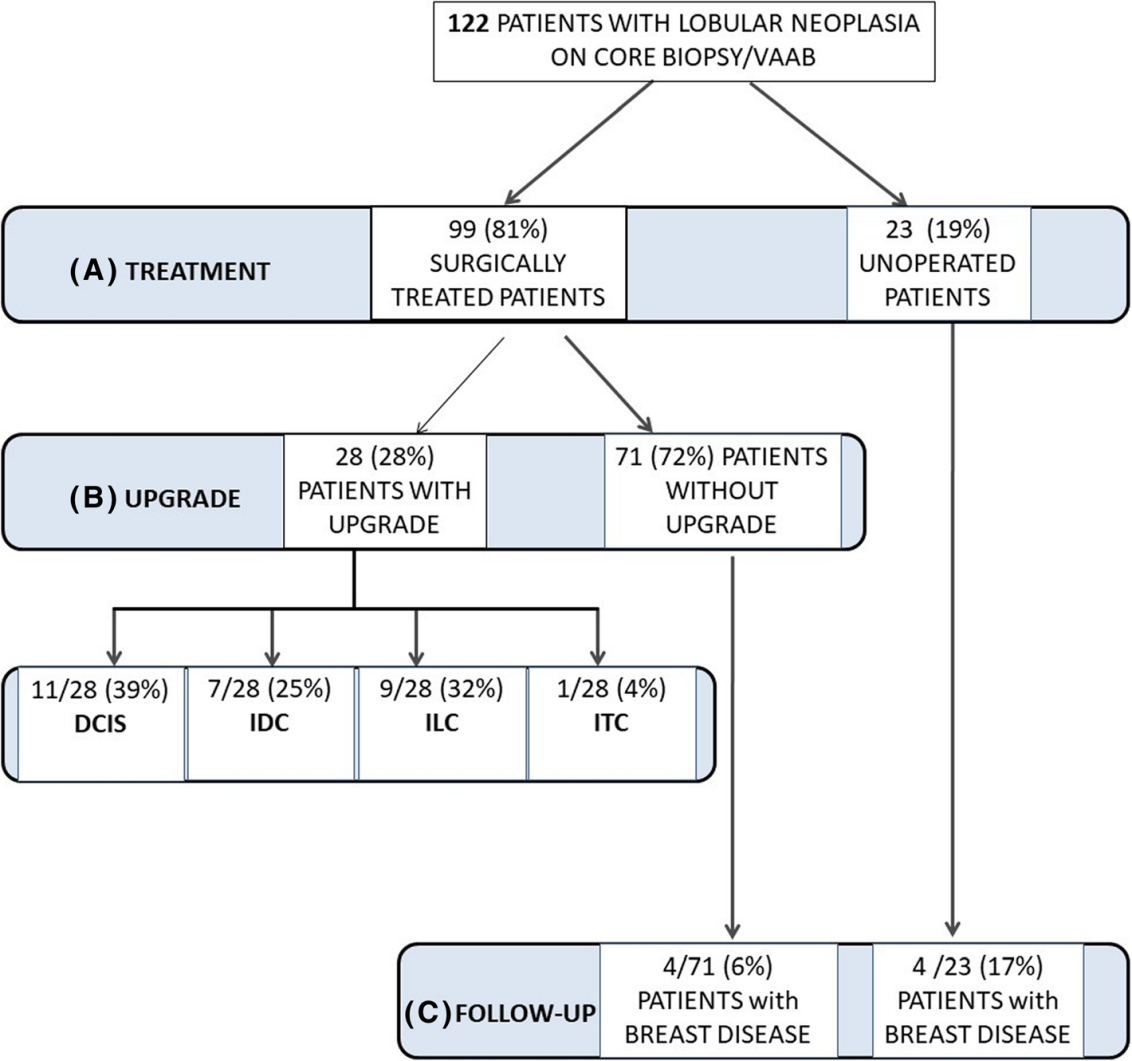
Study flowchart. Case series of 122 patients diagnosed with lobular neoplasia. Different treatment options (**a**). Upgrade at definitive diagnosis after surgical treatment (**b**). Occurrence of aggressive disease in operated and unoperated patients after a long follow-up (**c**). VAAB, vacuum-assisted breast biopsy; DCIS, ductal carcinoma in situ; IDC, invasive ductal carcinoma; ILC, invasive lobular carcinoma; ITC, invasive tubular carcinoma

A series of 122 consecutive female patients diagnosed with LN using CNB or VABB from January 1st, 2002, to December 31st, 2013, were collected from our electronic database system. All cases were followed at the Città della Salute e della Scienza Hospital in Turin, Italy. Women diagnosed with invasive carcinoma or ductal carcinoma in situ (DCIS) in the contralateral breast were excluded. A dedicated breast pathologist (IC) reviewed all the cases. The study was approved by the Research Ethics Committee for Human Biospecimen Utilization (Department of Medical Sciences—ChBU) of the University of Turin (n° 9/2019). Written consent was not required considering the retrospective nature of the study. The study was conducted in accordance with the Code of Ethics of the World Medical Association (Declaration of Helsinki). All cases were de-identified, and all clinical-pathological data were accessed anonymously, including age at diagnosis, family history of BC, previous breast surgical treatment, radiological diameter, and the Breast Imaging-Reporting and Data System (BI-RADS) [12]. As shown in Fig. 1, we stratified the study population according to the treatment option: surgically treated or follow-up only. In the first group, we collected the data on the upgrade status. Clinical follow-up was obtained and investigated for all patients.

In surgical cases, we obtained definitive histological diagnosis and subsequent treatment history (hormone therapy, radiotherapy, chemotherapy). In cases where the final histology found DCIS or invasive carcinoma, pathological features such as histotype, histological grade, vascular invasion, lymph nodal status, hormonal receptor expression, proliferation index (evaluated by Ki67), and HER2 assessment were collected.

### Statistical analysis

Statistical analyses were performed using the Stata 13.0 statistical software (StataCorp, College Station, TX, USA.). We used the Pearson chi-square test (*χ*^2^) test and Student’s *t* test to analyze the differences in the distribution of categorical and continuous variables. Univariate logistic regression analysis was used to calculate the odds ratios (ORs).

Analyses were conducted with a 95% confidence interval (CI), and a *p* value of 0.05 was considered statistically significant. All statistical tests were two-tailed.

## Results

### Patient characteristics and treatment approach

As shown in Table 1, 44/122 patients were diagnosed with ALH, 63/122 with LCIS, and 15/122 with HG-LN. The median medical follow-up was 9.5 years (range 6.4–12.1).

**Table 1:** Clinico-pathological features of the study population according to histological diagnosis (ALH, LCIS, HG-LN)

Clinico-pathological features		ALH (44)	LCIS (63)	HG-LN (15)	Total (122)	*p* value
Median age at diagnosis (interval)		54 (39–77)	52 (36–74)	56 (39–76)	54 (36–77)	0.714
Family history for BC	No	25 (56.9%)	41 (65%)	10 (66.7%)	76 (62.3%)	0.922
	Yes	15 (34.2%)	17 (27%)	4 (26.6%)	36 (29.5%)	
	Missing	4 (9%)	5 (8%)	1 (6.7%)	10 (8.2%)	
Previous breast surgery	No	30 (68.1%)	46 (73%)	11 (73.3%)	87 (71.3%)	0.919
	Yes	9 (20.5%)	13 (20.6%)	3 (20%)	25 (20.5%)	
	Missing	5 (11.4%)	4 (6.3%)	1 (6.7%)	8 (8.2%)	
Diagnostic procedure	Core needle biopsy	25 (56.8%)	27 (42.9%)	9 (60%)	61 (50%)	0.252
	Vacuum-assisted biopsy	19 (43.2%)	36 (57.1%)	6 (40%)	61 (50%)	
Radiological features	Microcalcifications	35 (79.6%)	45 (71.5%)	10 (66.7%)	90 (73.8%)	0.456
	Opacity	7 (15.9%)	12 (19%)	2 (13.3%)	21 (17.2%)	
	Architectural distortion	2 (4.5%)	6 (9.5%)	3 (20%)	11 (9%)	
Radiological diameter (mm)	< 10	18 (41%)	33 (52.3%)	6 (40%)	57 (46.7%)	0.507
	10–20	14 (31.8%)	17 (27%)	3 (20%)	34 (27.9%)	
	> 20	6 (13.6%)	3 (4.8%)	2 (13.4%)	11 (9%)	
	Missing	6 (13.6%)	10 (15.9%)	4 (26.6%)	20 (16.4%)	
BI-RADS category	BI-RADS 3	32 (72.7%)	43 (68.3%)	6 (40%)	81 (66.4%)	0.001
	BI-RADS 4	11 (25%)	13 (20.6%)	6 (40%)	30 (24.6%)	
	BI-RADS 5	0	1 (1.6%)	3 (20%)	4 (3.3%)	
	Missing	1 (2.3%)	6 (9.5%)	0	7 (5.7%)	
Associated lesions	No	28 (63.6%)	43 (68.2%)	13 (86.7%)	84 (68.9%)	0.248
	Yes	16 (36.4%)	20 (31.8%)	2 (13.3%)	38 (31.1%)	

The median age was 54 years (range 36–77 years). Thirty-six (29.5%) patients had a family history of BC. Previous breast surgery was reported in 25 (20.5%) patients. LN presented with radiological calcifications in most of the cases (73.8%), followed by tumor mass (17.2%) and architectural distortion (9%). The radiological diameter of the lesions was less than 10 mm in half of the cases (46.7%), and only 9% of the patients presented with lesions greater than 20 mm. CNB was performed in 50% of the cases, and VABB in the other half. BI-RADS categorization significantly correlated to LN grading; BI-RADS 4 and BI-RADS 5 typically indicated HG-LN diagnosis compared with LCIS or ALH (*p* = 0.001). In 31.1% of the cases, LNs were associated with benign lesions: flat epithelial atypia, atypical hyperplasia, sclero-elastotic lesions, papilloma, or fibroadenoma.

**Fig. 2:**
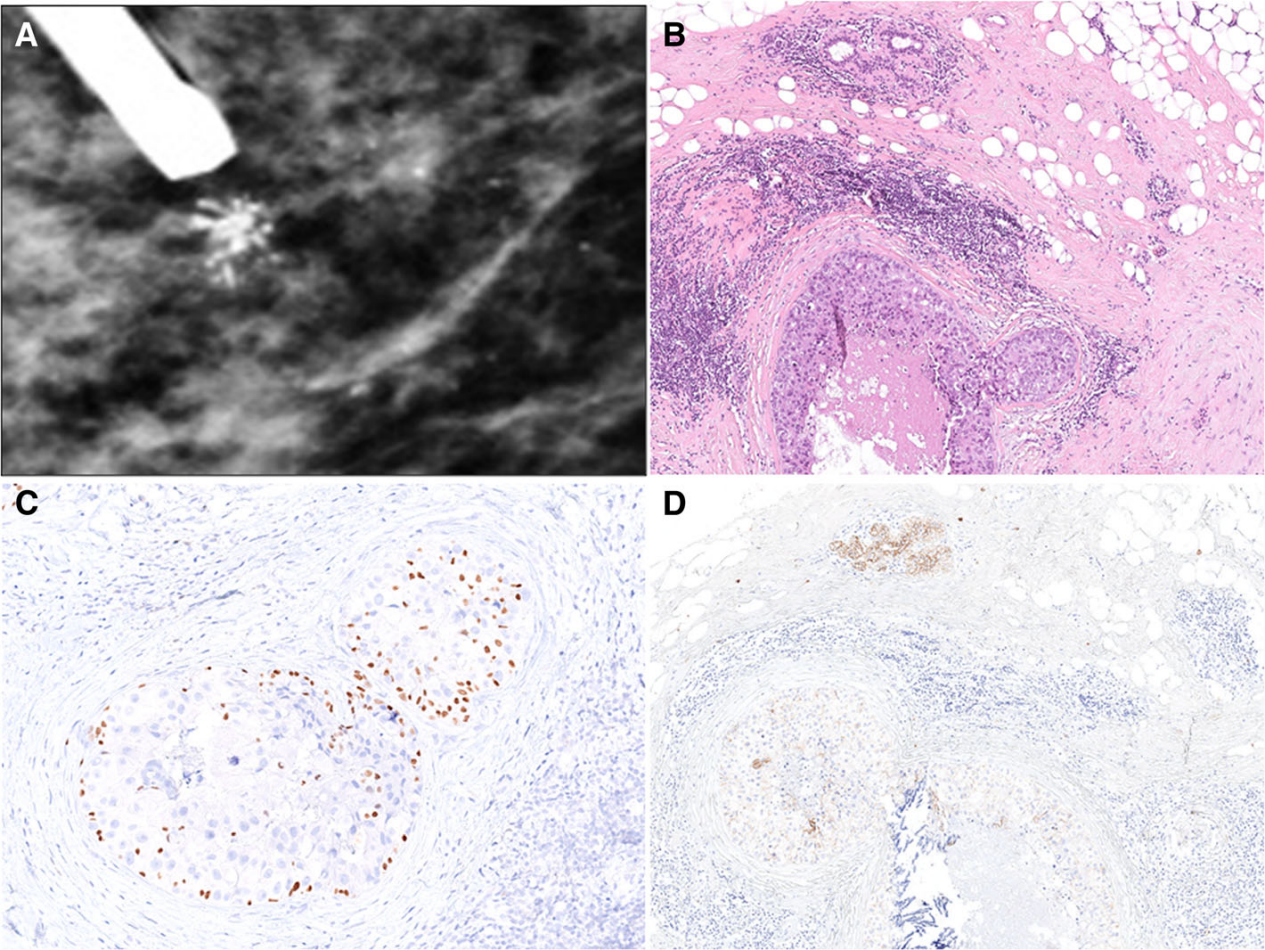
A representative case of lobular neoplasia. Vacuum-assisted biopsy of a microcalcifications cluster classified as BI-RADS R5 (**a**). Histologic appearance of corresponding pleomorphic lobular carcinoma in situ with areas of comedo-necrosis and periductal infiltrating lymphocytes (hematoxylin and eosin staining) (**b**, × 100). Immunohistochemical analyses of p63 (**c**, × 150). Myoepithelial layer with internal control of normal breast tissue and E-cadherin showing almost complete absence of staining (**d**, × 100)

Of the 122 patients, 99 (80.5%) underwent surgery, and 23 (19.5%) had observation with follow-up without therapy (Supplementary Table 1 and Fig. 1a). All patients classified as BI-RADS 5 and/or diagnosed with HG-LN received surgery. There were no other significant differences between these two groups (Supplementary Table 1). A representative radiological, histopathological, and immunohistochemical characteristic of a LN case is shown in Fig. 2.

### Surgically treated patients: characteristics and histological upgrade

Of the 99 surgically treated patients, 27 (27.2%) had a preoperative diagnosis of ALH, 57 (57.6%) of LCIS, and 15 (15.2%) of HG-LN (Supplementary Table 1). As shown in Table 2 and Fig. 1b, 28 (28.3%) lesions—3 ALH, 17 LCIS, and 8 HG-LN (*p* = 0.013)—were upgraded to an invasive carcinoma or DCIS at a definitive diagnosis. Univariate analysis revealed the variables significantly related to the risk of an upgraded lesion: (i) age ≥ 54 years (OR 4.01, CI 1.42–11.29, *p* = 0.009), (ii) BI-RADS categories 4 and 5 (OR 3.76, CI 1.37–10.1, *p* = 0.010), and (iii) preoperative diagnosis of HG-LN (OR 8.76, 1.82–42.27, *p* = 0.007) (Table 3).

**Table 2:** Frequency of histological upgrade and related variables among 99 patients who underwent excision

		Total (99)	Upgrade		*p* value
			No (71)	Yes (28)	
Median age at diagnosis (interval)		55 (36–74)	52 (36–74)	59 (39–76)	0.003
Age cutoff	< 54 years	46 (46.5%)	39 (54.9%)	7 (25%)	0.006
	≥ 54 years	53 (53.5%)	32 (45.1%)	21 (75%)	
Family history for BC missing 7	No	63 (63.6%)	46 (70.8%)	17 (63%)	0.528
	Yes	29 (36.4%)	19 (29.2%)	10 (27%)	
Previous breast surgery missing 7	No	72 (78.3%)	54 (83%)	18 (66.7%)	0.149
	Yes	20 (21.7%)	11 (17%)	9 (33.3%)	
Diagnostic procedure	Core needle biopsy	52 (52.5%)	36 (50.7%)	16 (57.1%)	0.563
	Vacuum-assisted biopsy	47 (47.5%)	35 (49.3%)	12 (42.9%)	
Radiological features	Microcalcifications	70 (70.7%)	51 (71.8%)	19 (67.9%)	0.722
	Mass	20 (20.2%)	13 (18.3%)	7 (25%)	
	Architectural distortion	9 (9.1%)	7 (9.8%)	2 (7.1%)	
Radiological diameter (mm) missing 16	< 10	47 (56.7%)	36 (60%)	11 (47.9%)	0.61
	10-20	29 (34.9%)	20 (33.3%)	9 (39.1%)	
	> 20	7 (8.4%)	4 (6.7%)	3 (13%)	
BI-RADS category missing 7	BI-RADS 3	65 (70.7%)	51 (78.5%)	14 (51.9%)	0.03
	BI-RADS 4	23 (25%)	12 (18.5%)	11 (40.7%)	
	BI-RADS 5	4 (4.3%)	2 (3%)	2 (7.4%)	
Biopsy histology	ALH	27 (27.2%)	24 (33.9%)	3 (10.7%)	0.013
	LCIS	57 (57.6%)	40 (56.3%)	17 (60.7%)	
	HG-LN	15 (15.2%)	7 (9.8%)	8 (28.6%)	
Associated lesions	No	66 (66.6%)	45 (63.4%)	21 (75%)	0.296
	Yes	33 (33.3%)	26 (36.6%)	7 (25%)

**Table 3:** Univariate logistic regression analysis of upgrade risk

Upgrade		OR	CI	*p*
Age	≥ 54 years	4.01	1.42–11.29	0.009
Family history for BC	No vs yes	0.76	0.35–1.68	0.504
Previous breast surgery	No vs yes	1.16	0.53–2.54	0.702
Diagnostic procedure	Core needle biopsy vs vacuum-assisted biopsy	0.74	0.29–1.86	0.524
Radiological features	Mass	1		
	Architectural distortion	0.52	0.08–3.36	0.496
	Microcalcifications	0.62	0.20–1.94	0.415
Radiological diameter	<10	1		
	10–20	1.77	0.58–5.38	0.309
	>20	3.00	0.57–15.8	0.196
BI-RADS category	BI-RADS 3 vs 4–5	3.76	1.37-10.1	0.010
Biopsy histology	ALH	1		
	LCIS	2.82	0.73–10.9	0.132
	HG-LN	8.76	1.82–42.27	0.007
Associated lesions	Yes vs no	0.62	0.23–1.71	0.363

At definitive histology, 11/28 patients showed DCIS, while invasive lobular (ILC), ductal (IDC), and tubular carcinoma were diagnosed in 9, 7, and 1 case, respectively (Supplementary Table 2). Most upgraded lesions had a diameter of < 20 mm, a positive estrogen (ER) and progesterone receptor (PgR) expression, and a low Ki67 index. Two cases showed lymph node involvement. Lymphovascular invasion was more frequent in patients preoperatively diagnosed with HG-LN (*p* = 0.028). One case exhibited HER2 overexpression (Supplementary Table 2).

In cases without an upgrade, a final diagnosis of LN was confirmed in 48/71 patients. The remainder received a benign diagnosis. Hormonal chemoprevention was administrated in 7/71 operated patients (6 LCIS and 1 HG-LN at final diagnosis), and 5 cases received radiotherapy (3 LCIS and 2 HG-LN at final diagnosis).

### Follow-up analysis

**Fig. 3:**
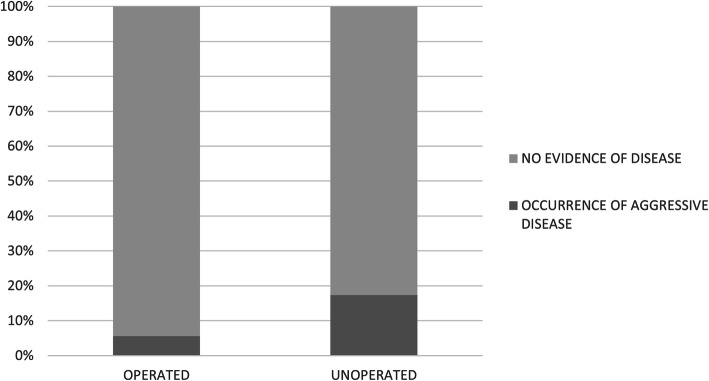
Diagram representation of aggressive disease occurrences in patients surgically treated and those who underwent observation only. In unoperated patients, 4/23 (17.4%) developed an aggressive disease

Follow-up data (Fig. 1c) for non-operated patients and those without post-surgical upgraded lesions showed that 8/94 (8.5%) later manifested an ipsilateral malignant lesion (3 ILC, 3 IDC, and 2 DCIS). The incidence of later malignancy for surgically treated patients was 4/71 (5.6%). Of those who were not surgically treated, 4/23 (17.4%) developed later malignancy found during follow-up (Fig. 3). No significant differences were observed between the groups of patients with and without recurrent disease.

## Discussion

In breast pathology, LN still represents an uncomfortable diagnosis due to the uncertainty of patient management. LN has been reclassified and renamed several times over the two last decades, and to date, this neoplasia is staged differently according to AJCC [7] or UICC [8]. Overall, these diverse terminologies complicate the clinical management decisions. The present case series confirms the clinical treatment uncertainty; patients with LN were managed with heterogeneous approaches (surgery or observation) mainly based on BI-RADS and LN grade assessment. The only cases consistently treated with surgery were the lesions with BI-RADS category 5 and/or with HG-LN. The preoperative biopsy technique (CNB or VABB) did not appear to influence upgrade at definitive histology. Our study showed that aggressive disease during follow-up occurred in 17% of non-operated patients regardless of known clinicopathological characteristics. Stratifying appropriate clinical management is important due to the risk of LN becoming an aggressive disease, which ranges from approximately 1 to 30% depending on the study [2, 10, 11]. This variability may be due to the different inclusion criteria (some studies, like ours, include HG-LN) and the variability in the number of samples obtained using CNB or VABB.

Our case series revealed that nearly 30% of surgical cases resulted in upgraded histology. BI-RADS categories 4–5, histological grading of LN at biopsy, and age ≥ 54 years were statistically associated with malignancy at definitive surgery. These data confirm the need for a multidisciplinary approach and a careful radio-pathological study before treatment decisions. The TBCRC 020 trial found that only 1% of LCIS and/or ALH on CNB with concordant imaging examination were upgraded on surgical samples, suggesting that surgical excision is not indicated in these cases when accurately diagnosed [13]. In line with this, European international recommendations [14] recently proposed to reserve open surgery for LN, mainly in cases of radio-pathological discordance [4, 15]. The LN WHO classification system is closely related to the risk of upgrade. Hussain et al. described the overall association to aggressive disease as 19%, 32%, and 41% in low, intermediate, and HG-LN, respectively [16]. In the study by Bianchi et al., the upgrade rate was 14.4% for ALH and 20.3% for LCIS [17].

Particular attention should be paid to HG-LN characterized by necrosis, microcalcifications, and pleomorphism (namely P-LCIS or florid LCIS). These lesions were recently described by Foschini et al. with an upgrade rate of almost 44% [18]. Accordingly, the present series showed that the upgrade rate of these lesions was 50%, confirming that excision is mandatory.

In line with our findings, the typical median age at LN diagnosis ranges from 50 [19] to 54 years [20]. In our series, 39.6% of patients > 54 years old had an upgrade at definitive histology compared to 15.2% of younger patients (*p* = 0.006). A possible explanation of this finding may be that, although LN mainly develops in premenopausal women [20–22], it is characterized by a long disease course. Thus, the lesions found in older patients are prone to upgrade into a more aggressive disease.

Although the prognosis of LN is heterogeneous, several studies suggested that 6–8% of operated patients may develop a carcinoma during follow-up [23, 24]. Our study found a similar rate: 8.5% of post-surgical patients developed more aggressive ipsilateral disease during the follow-up period (9-year median follow-up). Of the non-surgical, observational cases, 4 patients developed a more aggressive ipsilateral disease. This means that nearly 20% of untreated women with LN developed a more aggressive disease during the median follow-up time of 9 years. Similarly, King et al. [19] reported that these patients reach a cumulative risk of 26% of developing carcinoma after 15 years and that the annual cancer development rate is 2% per year after LN diagnosis (2% per year over 9-year follow-up = ~ 18%).

The present study has some limitations that warrant consideration. Its retrospective nature limits the collection of follow-up data. Moreover, the data described here are obtained on a relatively small number of patients; hence, confirmation on a larger cohort may be required.

In conclusion, our results demonstrate that age, radiological assessment, and HG-LN are associated with the risk of upgraded histology at definitive surgery. Therefore, these features should be examined carefully and discussed in a multidisciplinary context before deciding on patient management. Finally, close surveillance is essential due to the long-term risk of breast cancer in unoperated patients.

## Supplementary Information

**Additional file 1: Table S1.** Clinico-radiological and pathological characteristics of operated and non-operated patients.

**Additional file 2: Table S2.** Histopathological features of the upgraded lesions.

## Data Availability

The datasets used and/or analyzed during the current study are available from the corresponding author on reasonable request.
