# Supplementary material for: The lobular neoplasia enigma: management and prognosis in a long follow-up case series
Source: World J Surg Oncol. 2021 Mar 18;19:80. doi: 10.1186/s12957-021-02182-w (PMC7976718; doi:10.1186/s12957-021-02182-w)
Supplement: Supplementary file 1 — Additional file 1: Table S1. Clinico-radiological and pathological characteristics of operated and non-operated patients. [file 12957_2021_2182_MOESM1_ESM.docx]

**Supplementary Table 1. Clinico-radiological and pathological characteristics of operated and non-operated patients.**

|  | Breast Surgery | | p-value |
| --- | --- | --- | --- |
|  | **Yes**  **99 (80.5%)** | **No**  **23 (19.5%)** |  |
| Median Age | **55 (36-74)** | **55 (36-77)** | **/** |
| Family History  No  Yes  Missing | **63**  **29**  **7** | **15**  **8**  **/** | **ns*** |
| Previous breast surgery  No  Yes  Missing | **72**  **20**  **7** | **20**  **3**  **/** | **ns** |
| Radiological features  Microcalcifications  Opacity  Architectural distortion | **70**  **20**  **9** | **20**  **2**  **1** | **ns** |
| Radiological diameter  <10 mm  10-20 mm  >20 mm  Missing | **47**  **29**  **7**  **16** | **15**  **4**  **4**  **/** | **ns** |
| BI-RADS category  BI-RADS 3  BI-RADS 4  BI-RADS 5  Missing | **65**  **23**  **4**  **7** | **15**  **8**  **0**  **/** | **ns** |
| Histological diagnosis  ALH  LCIS  HG-LN | **27**  **57**  **15** | **17**  **6**  **0** | **0.0001** |

**ns: not significant*
