# Supplementary material for: The lobular neoplasia enigma: management and prognosis in a long follow-up case series
Source: World J Surg Oncol. 2021 Mar 18;19:80. doi: 10.1186/s12957-021-02182-w (PMC7976718; doi:10.1186/s12957-021-02182-w)
Supplement: Supplementary file 2 — Additional file 2: Table S2. Histopathological features of the upgraded lesions. [file 12957_2021_2182_MOESM2_ESM.docx]

**Supplementary Table 2: Histopathological features of the upgraded lesions.**

| **Pathological features** | | ALH | LCIS | HG-LN | TOTAL | p-value |
| --- | --- | --- | --- | --- | --- | --- |
| **Histotype** | **§DCIS** | 2 (75%) | 8 (47%) | 1 (25%) | 11 (39.3%) | 0.459 |
|  | **†IDC** | 1 (25%) | 3 (17.7%) | 3 (35%) | 7 (25%) |  |
|  | **^ILC** | 0 | 5 (29.4%) | 4 (50%) | 9 (32.1%) |  |
|  | **•TIC** | 0 | 1 (5.9%) | 0 | 1 (3.6%) |  |
| **pT** | **pTis** | 2 (75%) | 8 (47%) | 1 (25%) | 11 (39.3%) | 0.152 |
|  | **pTmic** | 0 | 1 (6%) | 3 (35%) | 4 (14.3%) |  |
|  | **pT1** | 1 (25%) | 8 (47%) | 4 (50%) | 13 (46.4%) |  |
| **pN** | **Nx** | 3 (100%) | 14 (82.3%) | 4 (50%) | 21 (75%) | 0.147 |
|  | **N0** | 0 | 3 (17.7%) | 2 (25%) | 5 (17.9%) |  |
|  | **N+** | 0 | 0 | 2 (25%) | 2 (7.1%) |  |
| ***Grade** | **G1** | 0 | 2 (22.3%) | 1 (14.2%) | 3 (17.6%) | 0.763 |
|  | **G2** | 1 (100%) | 7 (77.7%) | 5 (71.6%) | 13 (76.5%) |  |
|  | **G3** | 0 | 0 | 1 (14.2%) | 1 (5.9%) |  |
| ***ER** | **Positive** | 1 (100%) | 9 (100%) | 5 (71.6%) | 15 (88.2%) | 0.198 |
|  | **Negative** | 0 | 0 | 2 (28.4%) | 2 (11.8%) |  |
| ***PgR** | **Positive** | 1 (100%) | 7 (77.7%) | 4 (57.1%) | 12 (70.6%) | 0.532 |
|  | **Negative** | 0 | 2 (22.3%) | 3 (42.9%) | 5 (29.4%) |  |
| ***Lymphovascular invasion** | **Positive** | 0 | 1 (11.1%) | 2 (28.4%) | 3 (17.6%) | **0.028** |
|  | **Negative** | 1 (100%) | 8 (88.9%) | 5 (71.6%) | 14 (82.4%) |  |
| ***Ki67** | **<20%** | 1 (100%) | 9 (100%) | 5 (71.6%) | 15 (88.2%) | 0.198 |
|  | **≥20%** | 0 | 0 | 2 (28.4%) | 2 (11.8%) |  |
| ***HER2** | **Positive** | 0 | 0 | 1 (14.3%) | 1 (5.9%) | 0.468 |
|  | **Negative** | 1 (100%) | 9 (100%) | 6 (85.7%) | 16 (94.1%) |  |

*Estimated only for invasive BC

Abbreviations: §DCIS: ductal carcinoma in situ; †IDC: invasive ductal carcinoma; ^ILC: invasive lobular carcinoma; •ITC: invasive tubular carcinoma
